# The hidden link between late‐onset seizures and cerebral amyloid angiopathy: A case–control study

**DOI:** 10.1002/epi4.12976

**Published:** 2024-07-06

**Authors:** Oreste Marsico, Angelo Pascarella, Sara Gasparini, Lucia Manzo, Valentina Bova, Vittoria Cianci, Anna Mammì, Domenico Abelardo, Emilio Africa, Giuseppe La Torre, Antonio Armentano, Payam Tabaee Damavandi, Jacopo C. DiFrancesco, Umberto Aguglia, Edoardo Ferlazzo

**Affiliations:** ^1^ Department of Medical and Surgical Sciences Magna Græcia University of Catanzaro Catanzaro Italy; ^2^ Neurology Unit, Regional Epilepsy Centre Great Metropolitan "Bianchi‐Melacrino‐Morelli” Hospital Reggio Calabria Italy; ^3^ Unit of Neuroradiology Great Metropolitan “Bianchi‐Melacrino‐Morelli” Hospital Reggio Calabria Italy; ^4^ Department of Neurology Neurocenter of Southern Switzerland (NSI), EOC Lugano Switzerland; ^5^ Department of Neurology Fondazione IRCCS San Gerardo dei Tintori Monza Italy

**Keywords:** elderly, epilepsy, gradient echo, hemorrhage, MRI

## Abstract

**Objective:**

Epileptic seizures occurring in late adulthood often remain of unknown origin. Sporadic cerebral amyloid angiopathy (CAA) is a cerebral small vessel disease characterized by intracerebral hemorrhage, microhemorrhage and superficial siderosis, occurring mostly in elderly. This observational case–control study aimed to assess the occurrence of CAA in patients experiencing their first seizure in late adulthood.

**Methods:**

We enrolled consecutive patients aged ≥55 years presenting with late‐onset seizures (LOS) to the emergency departments or outpatient clinics of two Italian centers, from April 2021 to October 2022. Two age‐matched control subjects with neurological symptoms other than epileptic seizure were recruited for each enrolled case. All participants underwent brain MRI (1.5 Tesla) including blood‐sensitive sequences and were assessed for probable CAA diagnosis according to Boston criteria 2.0. Chi‐squared test was performed to evaluate group differences. Univariate logistic regression analysis tested the association between clinical variables and CAA.

**Results:**

We included 65 patients with LOS (27 females; mean age 72.2 ± 8.9 years) and 130 controls (49 females; mean age 70.3 ± 8.9 years). Diagnosis of probable CAA was achieved in 10.8% (7/65) of LOS patients and 2.3% (3/130) controls, with a statistically significant difference (*p* = 0.011). The OR for CAA in the LOS group was 5.2 as compared to the control group (95% CI = 1.3–20.6, *p* = 0.02).

**Significance:**

The frequency of CAA is significatively higher in patients with LOS as compared to other neurological diseases, suggesting that a portion of LOS of unknown or vascular origin are associated with CAA.

**Plain Language Summary:**

Late‐onset seizures (LOS) are very frequent in the elderly and often have no clear cause. Cerebral amyloid angiopathy (CAA) is a condition where amyloid proteins build up in the blood vessels of the brain, causing them to become weak and prone to bleeding. In this study, we explored the occurrence of CAA in people with LOS. We found that people with LOS were more likely to have a diagnosis of CAA than controls (i.e., people with other neurological diseases).

**Key points**

1. Late‐onset seizure (LOS) patients (*n* = 65) and controls (*n* = 130) underwent brain MRI with blood‐sensitive sequences.
2. The frequency of cerebral amyloid angiopathy (CAA) was significantly higher in patients with LOS as compared to controls.
3. LOS can be an onset manifestation of CAA.
4. Brain MRI with blood‐sensitive sequences should be performed in LOS patients.

## 1 INTRODUCTION

The occurrence of seizures and epilepsy significantly increases in elderly.^1^ Age‐related structural brain lesions, encompassing cerebrovascular disease, cerebral tumors and neurodegenerative disease, explain this high rate. In approximately half of late‐onset seizure (LOS) cases, the etiology remains unknown.^2^

Sporadic cerebral amyloid angiopathy (CAA) is a cerebrovascular disorder primarily affecting the elderly, characterized by the deposition of amyloid within small‐and medium‐sized blood vessels, arterioles and capillaries in the cerebral cortex and leptomeninges.^3^ CAA is recognized as a major risk factor for lobar intracerebral hemorrhages (ICH) mainly located in parietal, temporal and occipital lobes.^4^, ^5^, ^6^ Other clinical manifestations of CAA include insidious cognitive impairment as well as transient focal neurological episodes (TFNEs), known as *amyloid spells*.^7^ These are stereotypical attacks characterized by weakness, numbness, or jerking movements that progressively extend to other body regions, lasting 5–30 min.^8^ CAA‐related inflammation (CAA‐ri) is an additional and rare condition due to a perivascular or vascular inflammatory response to amyloid deposition,^9^ manifesting with rapidly progressive behavioral and cognitive decline, seizures, and headache.^10^, ^11^ Diagnosis of CAA is based on Boston criteria 2.0 that encompass both clinical findings (presentation with spontaneous ICH, TNFEs, or cognitive impairment or dementia) and MRI biomarkers (lobar ICH; lobar cerebral microbleeds, CMBs; cortical superficial siderosis, cSS; subarachnoid hemorrhages, SAH; “multispot” pattern white matter hyperintensities, WMHs; enlarged centrum semiovale perivascular spaces, CSO‐PVS).^12^ Current criteria do not include epileptic seizures. Nevertheless, two retrospective studies demonstrated the occurrence of seizures in about 20%–34% of patients with CAA, even as an index phenomenon.^13^, ^14^ CAA contribution to LOS remains uncertain and not well understood.

In the present study, we aimed to assess the frequency of CAA in patients with LOS as compared with age‐matched controls without seizures.

## 2 METHODS

### 2.1 Study population

In the present observational case–control study, consecutive patients aged ≥55 years presenting with new‐onset seizures or status epilepticus (SE) at the Unit of Neurology/Regional Epilepsy Centre of “Great Metropolitan Hospital” of Reggio Calabria and Unit of Neurology/Regional Epilepsy Centre, “Fondazione IRCCS San Gerardo dei Tintori” of Monza (Italy) from 1st April 2021 to 1st October 2022 were prospectively studied. The diagnosis of seizures or SE was ascertained by clinicians with expertise in epilepsy (SG, JDF, UA, VC, and EF) based on patients’ clinical and EEG data.^15^, ^16^, ^17^ Cases in which the diagnosis of epileptic seizures remained uncertain were not included. For each case, two age‐ and sex‐matched controls were recruited from patients with neurological symptoms other than seizures, referring to the Emergency Departments and Units of Neurology of the two involved centers. The exclusion criteria for both cases and controls were a history of previous epileptic seizures as assessed by expert epileptologists (SG, JDF, UA, VC, and EF) and contraindications to brain MRI.

The study adhered to the Strengthening the Reporting of Observational Studies in Epidemiology (STROBE) guidelines for reporting case–control studies.^18^

### 2.2 Procedure

For each subject, we recorded the following demographic and clinical data: age, sex, weight, smoking habit, presence of comorbidities (arterial hypertension, diabetes mellitus, dyslipidemia) and chronic use of anticoagulant or antiplatelet drugs, seizure/SE type (classified according to ILAE criteria)^15^, ^16^, ^17^ for cases and reason for seeking medical care for controls.

Enrolled subjects underwent cognitive assessment through the Italian version of the Montreal Cognitive Assessment (MoCA). As described in the literature, a MoCA score below of 22 (correct for age and years of education) was considered suggestive of cognitive impairment.^19^

All participants underwent brain MRI performed on a 1.5T machine (General Electric, Fairfield, CT). The neuroimaging protocol study included for all subjects axial T1‐weighted and T2‐weighted sequences, 3D fluid‐attenuated inversion recovery (FLAIR), diffusion‐weighted imaging (DWI), Gradient‐echo (GRE) and susceptibility‐weighted imaging (SWI) sequences. In the group of patients with LOS, MRI was performed within 30 days from seizure onset. MR images were reviewed by expert neuroradiologists (EA, AA, and GLT) blinded to the clinical context (i.e., presence or absence of seizures) to find out CAA neuroradiological markers (lobar CMBs, cSS, lobar ICHs, severe WMHs and enlarged CSO‐PVS).^12^

Following the Boston 2.0 criteria, a diagnosis of probable CAA was established for individuals exhibiting: at least one clinical finding (presentation with spontaneous ICH, TFNEs, or cognitive impairment/dementia) and (1) a minimum of two lobar hemorrhagic lesions (such as ICH, lobar CMBs, cSS, or SAH in any combination) or (2) a lobar hemorrhagic lesion plus one white matter feature (severe WMHs and enlarged CSO‐PVS).^12^

### 2.3 Statistical analyses

Continuous variables were expressed as mean ± standard deviation (SD), while categorical variables were indicated as per cent frequency. Differences between groups were calculated with a *t*‐test, Mann–Whitney test or chi‐squared test, as appropriate. The relationship between demographic, neuroradiological, clinical variables, and CAA was tested by the univariate logistic regression analysis, thus determining odds ratios (ORs) and 95% confidence intervals (CIs). A *p*‐value lower than 0.05 was considered statistically significant for all tests. All statistical analyses were performed via standard statistical packages (IBM SPSS Statistics 25.0 Corporation, Armonk, NY, USA).

### 2.4 Standard protocol approvals, registrations, and patient consents

The study was approved by the local Ethical Committee and was conducted following the Declaration of Helsinki. Each participant, their parents or legal representatives signed an informed consent form detailing the procedures. The data that support the findings of this study may be available on reasonable request.

## 3 RESULTS

**FIGURE 1:**
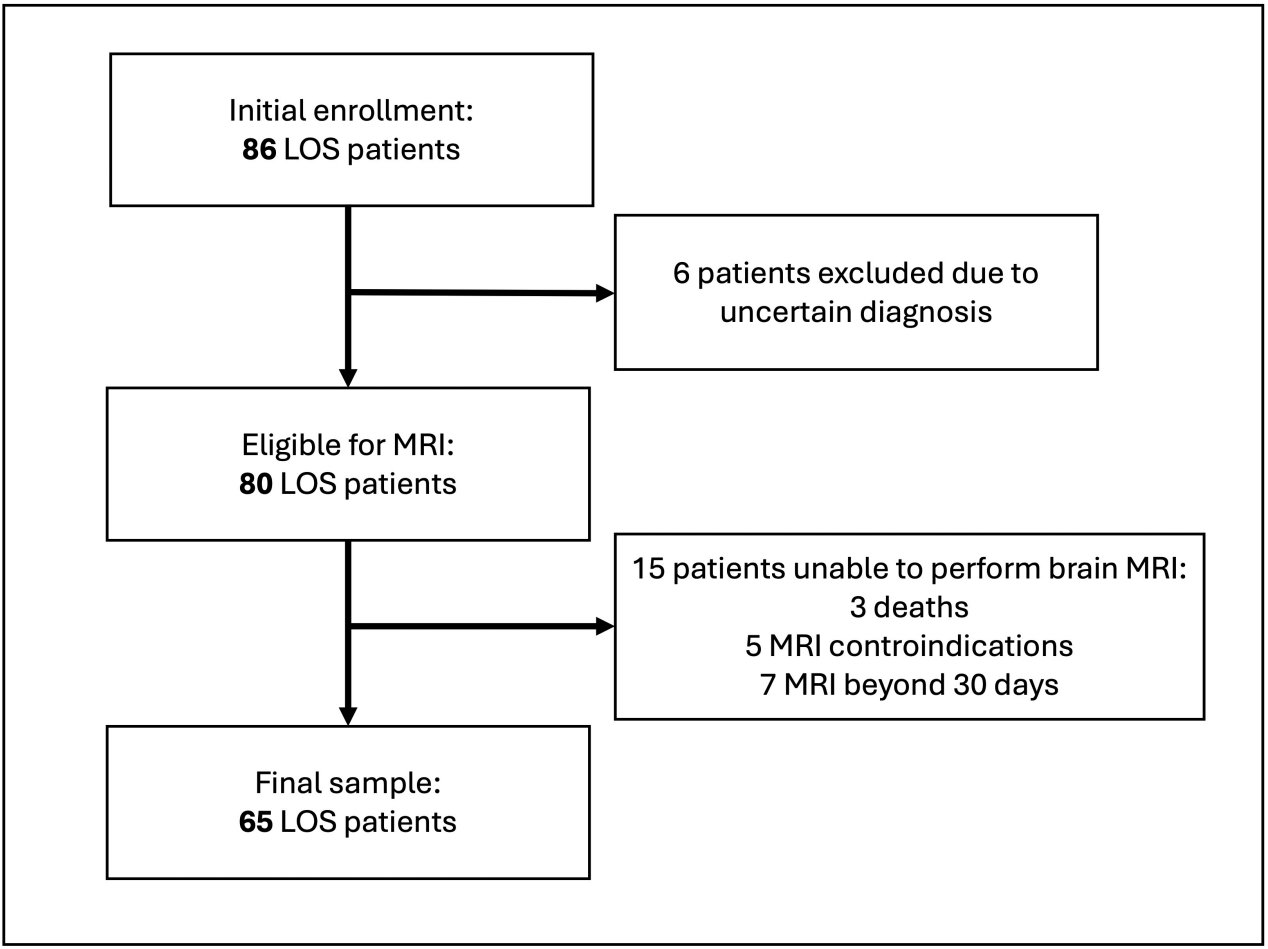
Study enrollment flowchart. Twenty‐one out of 86 initially enrolled patients were excluded due to uncertainty of LOS diagnosis (*n*: 6) or inability to perform brain MRI (*n*: 15).

A total of 86 patients with LOS were initially screened (see study flow chart, Figure 1). The final samples included 65 patients with LOS (27 females, mean age: 72.2 ± 8.9 years) and 130 control subjects (49 females, mean age: 70.3 ± 8.9 years). Demographic and clinical characteristics of the study population are reported in Table 1. The diagnosis of probable CAA was made in 7/65 patients of LOS group (10.8%) and in 3/130 controls (2.3%) (*χ* ^2^ = 6.45; *p* = 0.011) based on clinical and MRI findings. People with LOS had higher odds of having CAA as compared to control subjects (OR: 5.2; 95% CI = 1.3–20.6, *p* = 0.02).

**TABLE 1:** Clinical and demographic characteristics of the LOS and control group.

Patient's characteristics	LOS (*n*: 65)	Controls (*n*: 130)	*p*
Sex, female, *n* (%)	27 (41.5)	49 (37.7)	0.55
Age, years (± SD)	72.2 ± 8.9	70.3 ± 8.9	0.33
Smoking, *n* (%)	11 (16.9)	29 (22.3)	0.41
Comorbidity, *n* (%)			
Arterial hypertension	46 (70.7)	90 (69.2)	0.71
Diabetes mellitus	14 (21.5)	30 (23.1)	0.85
Hypercholesterolemia	22 (33.8)	51 (39.1)	0.51
Hypertriglyceridemia	8 (12.3)	29 (22.3)	0.10
Drugs, *n* (%)			
Anticoagulants	10 (15.4)	17 (13.0)	0.63
Antiplatelets	25 (38.5)	51 (39.2)	0.98

Abbreviations: LOS, late‐onset seizure; SD, standard deviation.

Persistent focal neurological deficits (limb weakness in 8 patients and speech disorders in 2 patients) were the clinical criterion for CAA diagnosis in all 10 subjects. Among CAA patients, 4 LOS subjects and 3 controls also exhibited cognitive impairment. Radiological signs consistent with lobar ICH were found in all patients diagnosed with CAA. Complete details about MRI characteristics of CAA subjects in both groups are reported in Table 2. Univariate logistic regression analysis did not show any association between specific MRI biomarkers of CAA and the occurrence of seizures. Table S1 illustrates comprehensive brain MRI findings in the overall study sample. Other overt causes for epileptic seizures (other than CAA) were not identified in all CAA cases. Additional data regarding the reason for neurological consultations for control subjects is provided in Table S2.

**TABLE 2:** MRI findings indicative of CAA in the LOS and control group.

	LOS	Control	*p*
CAA, *n* (%)	7/65 (10.8)	3/130 (2.3)	0.011^a^
Lobar ICH	7/7 (100)	3/3 (100)	
SAH	1/7 (14.3)	1/3 (33.3)	
CMB	3/7 (42.9)	1/3 (33.3)	
cSS	3/7 (42.9)	0	
CSO‐PVS/WMH	4/7 (57.1)	1/3 (33.3)	

Abbreviations: CAA, cerebral amyloid angiopathy; CMB, cerebral microbleeds; CSO‐PVS, centrum semiovale perivascular spaces; cSS, cortical superficial siderosis; ICH, intracerebral hemorrhage; LOS, late‐onset seizure; SAH, subarachnoid hemorrhage; WMH, white matter hyperintensities.

^a^ Significant statistical difference between the two groups.

Fifty‐seven out of 65 patients with LOS presented with a single seizure, whereas SE occurred in 8 patients. Details about seizure/SE semiology in LOS patients grouped according to CAA diagnosis are reported in Table 3. No difference in terms of clinical and EEG findings was seen between the LOS patients with or without CAA. Cognitive impairment, revealed by medical history and MoCA score <22, was detected in 27 LOS and 65 control subjects (Table 3). Among the 65 patients of the LOS group, MoCA examination was performed in 61 subjects. The presence of cognitive impairment was observed in 3/7 (42.8%) patients with CAA and 25/54 (46.3%) patients without CAA, without a statistically significant difference (*χ* ^2^: 0.03; *p* = 0.863).

**TABLE 3:** Clinical and EEG characteristics in the LOS group.

	CAA (*n*: 7)	Non‐CAA (*n*: 58)	*p*
Single seizure, *n* (%)	5/7 (71.4)	52/58 (89.6)	0.926
Focal	2/5 (40)	22/52 (42.3)	
Focal to bilateral TCS	2/5 (40)	18/52 (34.6)	
TCS	1/5 (20)	11/52 (21.1)	
Unknown	0	1/52 (1.9)	
Status epilepticus (SE), *n* (%)	2/5 (28.6)	6/58 (10.7)	0.108
Convulsive SE	1/2 (50)	2/6 (33.3)	
Focal motor SE	–	4/6 (66.7)	
Nonconvulsive SE	1/2 (50)	–	
Acute symptomatic seizure/SE, *n* (%)	6 (85.7)	28 (48.3)	0.094
EEG findings			
Epileptiform activity	4 (57.1)	29 (50)	0.718
Focal slowing	2 (28.6)	11 (19)	
Global slowing	1 (14.3)	10 (17.2)	
Normal interictal EEG	0	8 (13.8)	
Cognitive impairment^a^	3/7 (42.8)	25/54 (46.3)	0.863

Abbreviations: CAA, cerebral amyloid angiopathy; EEG, electroencephalogram; MoCA, Montreal Cognitive Assessment; NCSE, nonconvulsive status epilepticus; SE, status epilepticus; TCS, tonic–clonic seizure.

^**a**^ According to a MoCA <22; MoCA was performed in 7 CAA and 54 non‐CAA patients.

Lastly, examining MRI findings, we found two additional LOS patients with MRI markers satisfying neuroradiological criteria for probable CAA, but not fulfilling the diagnosis due to the lack of a clinical finding required by the Boston 2.0 diagnostic criteria.

## 4 DISCUSSION

In the present case–control study, we identified probable CAA in more than 10% of people experiencing their first seizure in late adulthood. This percentage was significantly higher as compared to matched subjects without seizures (with a five‐time risk for CAA in individuals with LOS). Currently, data about the association between CAA and seizures are scarce. To our knowledge, only two retrospective studies evaluated the frequency of epilepsy in CAA patients.^13^, ^14^ Tabaee Damavandi et al. found that 34% of 96 CAA patients developed seizures during a median follow‐up time of 13.5 months and they identified CAA‐ri and cSS as main risk factors for epilepsy.^13^ Freund et al. showed that roughly 20% of a cohort of 284 patients with CAA had seizures, presenting as the index event in more than one‐third of cases.^14^ To the best of our knowledge, this is the first case–control study aimed to investigate the frequency of CAA in patients with LOS. The different study design allowed us to identify the role of CAA as a risk factor for epileptic seizure in adulthood. Our study confirms the significant association between seizures and CAA and further highlights the possibility that seizures may represent an early manifestation of the disease.

Lobar ICH, cSS, and SAH may play a pathogenetic role due to the pro‐epileptogenic property of hemosiderin deposition on the cortex.^20^, ^21^, ^22^, ^23^, ^24^ Indeed, lobar ICH and cSS were reported as the main risk factors for seizures in CAA patients.^14^ Similarly, lobar ICH was demonstrable in all our CAA patients with seizures, even if regression analysis failed to demonstrate a specific MRI marker predicting the risk of seizure development, possibly due to the small sample size. Previous studies indicated that an inflammatory and immune‐mediated process may contribute to seizure development in CAA‐ri.^11^, ^25^ In our cohort, none of the LOS patients exhibited CAA‐ri and no MRI markers showed a statistically significant association with seizures.

LOS patients constitute a unique population expected to grow with population aging.^2^, ^26^, ^27^ In most cases, LOS are due to a cerebrovascular disease which may be undiagnosed at seizure onset.^28^, ^29^ Noteworthy, the ILAE Neuroimaging Task Force identified a set of recommended sequences including millimetric 3D T1, FLAIR and high‐resolution 2D submillimetric T2 images in the Harmonized Neuroimaging of Epilepsy Structural Sequences protocol. Blood‐sensitive sequences are not routinely recommended except in patients with suspected tumors, vascular malformations, or infectious processes.^30^ Brain MRI with blood‐sensitive sequences might improve the diagnosis of LOS allowing for a more precise delineation of cerebrovascular disease.^28^, ^31^, ^32^, ^33^ Antiplatelet or anticoagulant treatments should be carefully considered in CAA patients due to the combined risk of ICH and ischemic lesions.^34^

According to Boston criteria 2.0, a diagnosis of probable CAA strictly requires distinctive neuroradiological markers and specific clinical presentation, not encompassing epileptic seizures.^12^ Hence, we were unable to achieve the diagnosis of CAA in 2 out of 9 patients with seizures not showing clinical signs of major hemorrhages, cognitive impairment or TNFEs. This finding, along with the results of the previous studies,^13^, ^14^ suggests that it might be advisable to consider epileptic seizures as a clinical symptom of CAA in a future revision of diagnostic criteria. Our study has some limits. First, the sample size is apparently limited; however, considering the characteristics of study population (patients presenting first seizure in late adulthood), the strict inclusion criteria (MRI within 30 days from seizure onset), and the study design with prospective enrollment, the number of participants seems suitable. Secondly, the diagnosis of CAA was posed on clinical and radiological criteria, without a definitive histopathological confirmation. Brain biopsy is an invasive and complex procedure not easily feasible in the clinical setting. Nevertheless, we chose to focus on “probable” CAA diagnosis only, not considering patients with possible CAA, to increase our diagnostic accuracy and strengths of the results.

## 5 CONCLUSION

This study shows that the frequency of CAA is significatively higher in patients with LOS compared to other neurological diseases. MRI with blood‐sensitive sequences should be performed in LOS of unknown or vascular origin.

## AUTHOR CONTRIBUTIONS

*Conceptualization*: Edoardo Ferlazzo, Sara Gasparini, Umberto Aguglia. *Methodology*: Sara Gasparini, Edoardo Ferlazzo Oreste Marsico, Angelo Pascarella. *Formal analysis and investigation*: Oreste Marsico, Angelo Pascarella, Domenico Abelardo. *Data collection*: Oreste Marsico, Angelo Pascarella, Lucia Manzo, Valentina Bova, Vittoria Cianci, Anna Mammì, Emilio Africa, Giuseppe La Torre, Antonio Armentano, Payam Tabaee Damavandi. *Writing*—*original draft preparation*: Oreste Marsico, Angelo Pascarella. *Writing*—*review and editing*: Oreste Marsico, Angelo Pascarella, Edoardo Ferlazzo, Sara Gasparini, Jacopo C. Difrancesco, Umberto Aguglia. *Resources*: Umberto Aguglia. *Supervision*: Edoardo Ferlazzo, Sara Gasparini, Umberto Aguglia.

## CONFLICT OF INTEREST STATEMENT

Oreste Marsico, Angelo Pascarella, Sara Gasparini, Lucia Manzo, Vittoria Cianci, Valentina Bova, Anna Mammì, Domenico Abelardo, Emilio Africa, Giuseppe La Torre, Antonio Armentano, Payam Tabaee Damavandi, Jacopo C. DiFrancesco and Umberto Aguglia report no disclosures. Edoardo Ferlazzo received speaker honoraria from UCB, EISAI, Angelini. We confirm that we have read the Journal's position on issues involved in ethical publication and affirm that this report is consistent with those guidelines.

## ETHICS STATEMENT

The study was performed in accordance with the ethical standards laid down in the 1964 Declaration of Helsinki and its later amendments.

## PATIENT CONSENT STATEMENT

Patients have given informed consent, allowing publication of the material, with the understanding that it will not be used for any other purposes than medical publication.

## Supporting information


Table S1.
Table S2.
