# Supplementary material for: The hidden link between late‐onset seizures and cerebral amyloid angiopathy: A case–control study
Source: Epilepsia Open. 2024 Jul 6;9(5):1723–30. doi: 10.1002/epi4.12976 (PMC11450647; doi:10.1002/epi4.12976)
Supplement: Supplementary file 1 — Table S1. Table S2. [file EPI4-9-1723-s001.docx]

**Suppl. Table 1. MRI features in LOS subjects and control.**

|  | **LOS (n: 65)** | **Controls (n: 130)** | |
| --- | --- | --- | --- |
| Cerebral Amyloid Angiopathy, n (%) | 7 (10.8) | | 3 (2.3) |
| Ischemic stroke, n (%) | 27 (41.5) | | 44 (33.8) |
| Deep Hemorrhage, n (%) | 4 (6.1) | | 7 (5.4) |
| Tumor, n (%) | 7 (10.8) | | 10 (7.7) |
| Autoimmune encephalitis lesions, n (%) | - | | 1 (0.8) |
| Infectious encephalitis lesions, n (%) | 6 (9.2) | | 10 (7.7) |
| Demyelinating lesions, n (%) | - | | 9 (6.9) |
| Diffuse cortical atrophy, n (%) | 3 (4.6) | | 6 (4.6) |
| Traumatic lesions, n (%) | 1 (1.6) | | - |
| Normal, n (%) | 10 (15.4) | | 40 (30.8) |

**Suppl. Table 2. Clinical reasons for MRI investigations in controls**

|  |  |  | **Controls (n: 130)** |
| --- | --- | --- | --- |
| Acute onset of focal neurological signs, n (%) |  |  | 54 (41.5) |
| Headache, n (%) |  |  | 20 (15.4) |
| Dizziness, n (%) |  |  | 12 (9.2) |
| Suspected encephalitis, n (%) |  |  | 11 (8.5) |
| Follow-up brain tumor, n (%) |  |  | 10 (7.7) |
| Suspected multiple sclerosis, n (%) |  |  | 9 (6.9) |
| Movement disorders, n (%) |  |  | 8 (6.2) |
| Cognitive impairment, n (%) |  |  | 6 (4.6) |
